# Familial History of Diabetes is Associated with Poor Glycaemic Control in Type 2 Diabetics: A Cross-sectional Study

**DOI:** 10.1038/s41598-017-01527-4

**Published:** 2017-05-03

**Authors:** Ming Wu, Jinbo Wen, Yu Qin, Hailong Zhao, Xiaoqun Pan, Jian Su, Wencong Du, Enchun Pan, Qin Zhang, Ning Zhang, Hongyan Sheng, Chunlan Liu, Chong Shen

**Affiliations:** 1 Department of Non-communicable Chronic Disease Control, Jiangsu Provincial Center for Disease Control and Prevention, Nanjing, 210009 China; 2 Department of Epidemiology, School of Public Health, Nanjing Medical University, Nanjing, 211166 China; 3 Department of Chronic Disease Prevention and Control, Huai’an City Center for Disease Control and Prevention, Huai’an, 223001 China; 4 Changshu County Center for Disease Control and Prevention, Suzhou, 215500 China

## Abstract

To investigate the association of familial history (FH) of diabetes with the glycaemic control status of patients with type 2 diabetes (T2D), a cross-sectional study using stratified cluster sampling was conducted with 20,340 diabetic patients in Jiangsu, China. In total, 21.3% of the subjects reported a FH of diabetes. Patients with a FH of diabetes showed a higher risk of poor glycaemic control (59.7%) than those without a diabetic FH (49.8%), with an odds ratio (OR) of 1.366 (*P* < 0.001). Glycaemic control status did not significantly differ between the T2D patients with parental FH and those with sibling FH. Compared with patients with paternal FH, patients with maternal FH had a higher risk of poor glycaemic control (OR = 1.611, *P* = 0.013). Stratified analyses showed that a FH of diabetes was significantly associated with poor glycaemic control among T2D patients with a low education level (*P* < 0.05). In the <60 years old, overweight, and low level of physical activity groups, patients with a maternal history of diabetes showed a higher risk of poor glycaemic control than those without a FH (*P* < 0.05). In conclusion, FH of diabetes, especially a maternal history, had an independently adverse effect on the glycaemic control of T2D patients.

## Introduction

In recent decades, non-communicable diseases (NCDs) have contributed substantially to the increased mortality rate and have massively increased the global burden of disease^1, 2^. Diabetes, as one of the NCDs of most concern, was shown to have an overall prevalence of 11.6% in China in 2010^1^. It is an understatement to say that the current prevalence of diabetes merely outweighs the figure in 1980, as it is greater by ten-fold^3–6^. While diabetes and its complications pose threats to patients’ health, the associated medical expenditures inevitably cause a large financial burden for the patients, families, and society as a whole^6–8^.

A familial history (FH) of diabetes has extensively been reported to be associated with an earlier onset of diabetes among patients^9–13^ and with a higher incidence of diabetes in the general population^14–17^. People diagnosed with early-onset type 2 diabetes (T2D) were found to face more difficulties in managing their glycaemic control and to be more likely to experience microvascular complications^18–22^. However, the association between a FH of diabetes and glycaemic control of patients with diabetes remains controversial^13, 23–26^.

Several factors, including the maternally determined environment (intrauterine and early postnatal), genomic imprinting, and nutritional/lifestyle influences, can all contribute to the incidence of diabetes and hyperglycaemia^27–29^. However, the mechanisms underlying the relationships are poorly understood. An improved understanding of the role of diabetic FH in the development of diabetes may help explain the association between a FH of diabetes and glycaemic control of T2D patients. Using the number of affected first-degree relatives (FDRs) and second-degree relatives (SDRs), Valdez R. *et al*. found that the odds of developing diabetes among individuals with moderate (only one FDR and one SDR, one FDR, or two SDRs) and high familial risk (at least one FDR and two SDRs or two FDRs) were 2.3 and 5.5 times higher, respectively, than the odds among those with an average risk (none or only one SDR)^30^. However, few investigations have investigated the relevance of a FH of diabetes and glycaemic control status in the Chinese population. In the present study, we examined the relationship between a FH of diabetes and the glycaemic control of T2D patients using a combined evaluation of fasting plasma glucose (FPG) and haemoglobin A1c (HbA1c).

## Methods

### Subjects

A cross-sectional study using stratified cluster sampling was conducted in 65 townships of three areas (Changshu in Suzhou City and Huaiyin and Chuzhou in Huai’an City, Jiangsu Province, China), and a total of 39,564 patients with T2D were targeted. Townships operating solely within the health care programme of the National Basic Public Health Service (2012) were included. From the 44 selected townships, 23,240 of the T2D patients were recruited for this investigation. According to the criteria established by the American Diabetes Association (ADA) in 2010, T2D patients were defined as having FPG levels ≥7.0 mmol/L or a self-reported T2D history without type 1 diabetes. A total of 20,340 subjects consented to complete a standardized epidemiology questionnaire and physical examinations. All examinations were performed within two months (from Dec 2013 to Jan 2014). FPG and HbA1c measurements were collected from 20,015 and 19,997 of the T2D patients, respectively, and 19,992 of the T2D patients had both FPG and HbA1c data available. General characteristics of the participants are shown in Supplementary Table 1.

The study protocol was approved by the Ethics Board of the Jiangsu Provincial Centers for Disease Control and Prevention (No. 2013026). All patients were well informed about the study and provided written consent before participating; all methods were performed in accordance with the relevant guidelines and regulations.

### Questionnaire survey, anthropometric measurements, and biochemical indices detection

The investigators were trained and qualified in a standardized manner. The training contained all the relevant information and procedures for the questionnaire, anthropometric measurements, blood sample collection and processing, and quality control. The investigators were qualified after the training and prior to the formal investigation. Personal information on the T2D patients, including demographic characteristics, medical history, and lifestyle factors, was collected using a standardized questionnaire.

Anthropometric measurements, including height and body weight, were obtained by trained technicians. Body weight and height were measured twice in individuals ensuring that heavy clothes and shoes were removed before measurements, and the figures were rounded to the nearest 0.1 kg and 0.1 cm, respectively. Body mass index (BMI) was calculated as weight (kg)/height squared (m^2^).

Blood samples were collected 8 hours after the participant’s last meal or after an overnight fast. Fasting plasma glucose was assessed using the enzymatic method, and the whole blood HbA1c level was assessed using commercially available reagents from KingMed Diagnostics (Jiangsu Cultural Industrial Park, Nanjing, China).

### Definition

The outcome assessment indices for the glycaemic control of T2D were categorized as <7 mmol/L (controlled) and ≥7 mmol/L (uncontrolled) for FPG and <7% (controlled) and ≥7%^31^ (uncontrolled) for HbA1c. In addition, an evaluation of HbA1c and FPG combined was used to assess glycaemic control status. The level of glycaemic control was defined as “good”, “common” and “poor” when the criteria of HbA1c <7% (53 mmol/mol) and FPG <7 mmol/L were both met, when either criterion was met, and when neither was met, respectively.

Patients’ educational levels were classified into 4 categories as follows: “without formal education”, “primary”, “middle school”, and “high school and above”. Smoking habits were analysed by “yes” or “no” responses to the question, “Have you ever smoked 100 cigarettes in the past”? Drinking was defined as consuming at least once alcoholic drink per month, and drinking habits were categorized into “current”, “former” and “no drinking”. Subjects with a BMI of 18.5 to 23.9, 24 to 27.9, and ≥28 kg/m^2^ were defined as normal weight, overweight, and obese, respectively^32^. Duration of T2D was defined as the time between the survey and the time the patient was diagnosed with T2D. Physical activity included both dynamic behaviour and static behaviour. Dynamic behaviours represented physical activities at work, transportation, and leisure time. The intensity of activities was defined as vigorous or moderate during work and leisure time according to the duration and amount. The level of participants’ physical activity per day was then assessed by estimating the metabolic equivalent (MET). By its definition, the MET is a ratio of the working metabolic rate to a standard resting metabolic rate of 1.0 (4.184 kJ) · kg^−1^ · h^−1^, and 1 MET was considered the resting metabolic rate obtained in a sedentary setting^33^. The weekly time participants engaged in dynamic behaviours was divided by 7 to generate the average time of daily activity. For the purpose of rapid scoring, the following MET levels were assigned to each class of activity: sleep = 1 MET; static activity = 1.1 METs; moderate activity = 4 METs, and vigorous activity = 8 METs^34, 35^. In the stratified analysis, age (year) was categorized into <60 and ≥60 years old, and physical activity was classified as low (MET < 31.22) or high (MET ≥ 31.22) based on the median MET (31.22).

According to whether the patients’ father, mother, sibling, grandparents/maternal grandparents, uncles, and/or aunts were reported to have diabetes, the FH of diabetes was generally categorized into “parental”, “sibling”, and “other relatives”. Based on the combination of parental FH, sibling FH, and other relatives FH, patients were categorized into eight groups, and their average HbA1c and FPG level and glycaemic control status are shown in Supplementary Table 2. Patients with “parental only” history of diabetes were further classified into three groups: “maternal”, “paternal”, and “bi-parental”. The diabetes status of the patients’ spouses was also investigated.

### Statistical analysis

The mean (SD range) was calculated for continuous variables with a normal distribution, whereas those with a skewed distribution were described as the median [inter-quartile range (IQR)]. A chi-square (*χ* ^2^) test was used for the comparison of categorized variables. Student’s t-test and analysis of variance (ANOVA) were used to compare the normally distributed continuous variables, and Mann-Whitney *U* test was used to compare abnormally distributed data. Logistic regression analyses were performed, and the relative risk was estimated as the odds ratio (OR) and 95% confidence interval (CI). Stratified analyses were conducted by age, gender, education, BMI, antidiabetic treatment, and physical activity. Bonferroni correction was performed for multiple comparison tests. A two-tailed *P* value < 0.05 was considered to indicate statistically significance. All statistical analyses were performed using IBM SPSS Statistics 15.0 (SPSS, Inc, Chicago, USA) and Stata 12.0 (College Station, TX, USA).

## Results

### General characteristics of participants

The average age of the subjects was 63.35 ± 9.86 (years), and 39.2% of the subjects were male. Overall, 32.0% of T2D patients had a FPG level considered controlled (<7 mmol/L), and 41.9% were controlled based on HbA1c (<7%). With regard to glycaemic control according to both FPG & HbA1c, 25.7% of the patients achieved a good level, 22.4% were in the common level, and the remaining 51.9% were poorly controlled. A total of 917 patients were excluded for missing data regarding their FH of diabetes, and an additional 200 patients were excluded for missing information about details of their FH. As a result, 21.3% of the total 19,075 patients self-reported a FH of diabetes, and 18,875 individuals provided detailed information about their FH, in which the percentage of familial diabetes from their mother, father, and both parents represented 5.9%, 2.6%, and 0.8%, respectively (Supplementary Table 1).

### Demographic characteristics among glycaemic control groups

The demographic characteristics and FH of diabetes were listed according to glycaemic control status as determined by FPG and HbA1c levels (Table 1). Overall, the patients with glycaemic control in terms of HbA1c <7% and FPG <7 mmol/L had a slightly higher age but a shorter duration of T2D and lower BMI than those with HbA1c >7% and FPG >7 mmol/L (P < 0.001). Patients who were female, did not smoke, had a spouse with diabetes, did not receive antidiabetic treatment and had no FH of diabetes had a significantly higher proportion of controlled HbA1c <7% and FPG <7 mmol/L than their counterparts (P < 0.05). Current drinkers had a lower proportion (28.2%) of controlled FPG <7 mmol/L than those who never consumed alcohol (32.7%) (*P* < 0.001).

**Table 1:** Characteristics based on different glycaemic control status using HbA1c and FPG.

Variable*	HbA1c (%) category			FPG (mmol/L) category			HbA1c (%) & FPG (mmol/L)			
	<7 (n = 7990)	≥7 (n = 11085)	*P*	<7 (n = 6078)	≥7 (n = 12997)	*P*	Good (n = 4897)	Common (n = 4274)	Poor (n = 9904)	*P*
Age (year)	63.56 ± 9.72	62.98 ± 9.86	<0.001	63.83 ± 9.69	62.93 ± 9.84	<0.001	63.61 ± 9.85	63.83 ± 9.38	62.76 ± 9.94	<0.001
Gender										
Male	3010 (40.2)	4481 (59.8)	<0.001	2200 (29.4)	5291 (70.6)	<0.001	1759 (23.5)	1692 (22.6)	4040 (53.9)	<0.001
Female	4980 (43.0)	6604 (57.0)		3878 (33.5)	7706 (66.5)		3138 (27.1)	2582 (22.3)	5864 (50.6)	
Education										
Without formal education	3086 (43.6)	3987 (56.4)	<0.001	2413 (34.1)	4660 (65.9)	<0.001	1983 (28.0)	1533 (21.7)	3557 (50.3)	<0.001
Primary	2709 (41.5)	3818 (58.5)		1994 (30.6)	4533 (69.4)		1590 (24.4)	1523 (23.3)	3414 (52.3)	
Middle school	1431 (39.3)	2213 (60.7)		1060 (29.1)	2584 (70.9)		839 (23.0)	813 (22.3)	1992 (54.7)	
High school and above	735 (41.7)	1028 (58.3)		588 (33.4)	1175 (66.6)		465 (26.4)	393 (22.3)	905 (51.3)	
Smoking										
Yes	2049 (38.0)	3349 (62.0)	<0.001	1597 (29.6)	3801 (70.4)	<0.001	1264 (23.4)	1118 (20.7)	3016 (55.9)	<0.001
No	5941 (43.4)	7736 (56.6)		4481 (32.8)	9196 (67.2)		3633 (26.7)	3156 (23.1)	6888 (50.4)	
Drinking										
Yes	1339 (41.4)	1896 (58.6)	0.582	912 (28.2)	2323 (71.8)	<0.001	753 (23.3)	745 (23.0)	1737 (53.7)	0.010
Past	381 (40.7)	556 (59.3)		296 (31.6)	641 (68.4)		230 (24.5)	217 (23.2)	490 (52.3)	
No	6244 (42.1)	8602 (57.9)		4853 (32.7)	9993 (67.3)		3901 (26.3)	3295 (22.2)	7650 (51.5)	
Antidiabetic treatment										
Yes	4909 (32.8)	10042 (67.2)	<0.001	3429 (22.9)	11522 (77.1)	<0.001	2385 (16.0)	3568 (23.9)	8998 (60.2)	<0.001
No	2973 (74.6)	1014 (25.4)		2563 (64.3)	1424 (35.7)		2429 (60.9)	678 (17.0)	880 (22.1)	
Duration (year)	3.42 (1.42, 6.17)	5.75 (3.08, 10.67)	<0.001	3.00 (1.33, 5.83)	5.50 (2.92, 10.33)	<0.001	2.58 (1.17, 5.25)	4.67 (2.33, 8.58)	5.92 (3.17, 10.67)	<0.001
Spouse with diabetes										
Yes	374 (49.3)	385 (50.7)	<0.001	316 (41.6)	443 (58.4)	<0.001	268 (35.3)	154 (20.3)	337 (44.4)	<0.001
No	7532 (41.6)	10584 (58.4)		5697 (31.4)	12419 (68.6)		4579 (25.3)	4071 (22.5)	9466 (52.3)	
Familial history of diabetes										
Yes	1381 (33.9)	2689 (66.1)	<0.001	981 (24.1)	3089 (75.9)	<0.001	720 (17.7)	922 (22.7)	2428 (59.7)	<0.001
No	6609 (44.0)	8396 (56.0)		5097 (34.0)	9908 (66.0)		4177 (27.8)	3352 (22.3)	7476 (49.8)	
BMI (kg/m^2^)	25.11 ± 3.43	25.47 ± 3.48	<0.001	25.35 ± 3.58	25.32 ± 3.41	0.515	25.25 ± 3.53	25.13 ± 3.41	25.45 ± 3.44	<0.001
Physical activity (MET h/day)	31.40 (27.67, 37.10)	31.20 (27.62, 36.99)	0.065	31.23 (27.63, 36.43)	31.22 (27.67, 37.20)	0.393	31.35 (27.67, 36.79)	31.29 (27.67, 37.00)	31.20 (27.65, 37.10)	0.787

Continuous variables were presented as the means (SD), and categorized variables as numbers (percentages).

*P* values were from one-way analysis or Mann-Whitney *U* test for continuous variables and from chi-squared test for categorical variables.

*There were 68 missing values for education; 57 missing values for drinking; 137 missing values for antidiabetic treatment; 70 missing values for duration of diabetes; 27 missing values for BMI, which was calculated as weight in kilograms divided by height in metres squared; and 1232 missing values for physical activity.

The differential characteristics noted above were also observed among patients with “poor”, “common” and “good” levels of glycaemic control (Table 1). Thus, these factors were adjusted for when evaluating the association between FH of diabetes and glycaemic control.

### Glycaemic control status among the groups with a FH of diabetes

A lower proportion of patients with a FH of diabetes were under glycaemic control than those without a FH (P < 0.05) based on HbA1c (33.9% vs. 44.0%), FPG (24.1% vs. 34.0%), and HbA1c & FPG (17.7% vs. 27.8%) (Table 1).

The FPG and HbA1c levels and proportion of glycaemic control in terms of FPG, HbA1c and HbA1c & FPG did not significantly differ between the “parental and sibling”, “parental only”, and “sibling only” groups (*P* > 0.05, Table 2), and no significant difference in the proportion of those with glycaemic control based on FPG, HbA1c and HbA1c & FPG was observed between the parental and/or sibling, other relatives, and both parental and/or sibling and other relatives groups (*P* > 0.05, Supplementary Table 3). Meanwhile, glycaemic control according to HbA1c <7% increased gradually across the “bi-parental”, “maternal”, and “paternal” groups (25.9%, 33.4%, and 40.1%, respectively) (*P*_trend_ < 0.01), and the proportion of those with “good” glycaemic control based on HbA1c & FPG (15.3%, 16.5% and 21.9%, respectively) also increased across the three groups (*P*_trend_ < 0.01). By contrast, no significant difference in FPG or HbA1c levels was observed between the three groups (*P* > 0.05).

**Table 2:** Comparison of FPG, HbA1c and glycaemic control among type 2 diabetes patients with different FH of diabetes.

Variable	FH of diabetes					Parental history of diabetes				
	Parental and sibling (n = 481)	Parental only (n = 1226)	Sibling only (n = 1997)	*P*	*P*_trend_*	Bi-parental (n = 85)	Maternal (n = 794)	Paternal (n = 347)	*P*	*P*_trend_*
Age at diagnosis^†^	50.36 ± 8.76	49.43 ± 9.80	56.07 ± 9.48	<0.001	0.068	47.50 ± 8.64	49.34 ± 9.61	50.10 ± 10.44	0.082	0.028
FPG	9.52 ± 3.51	9.28 ± 3.34	9.14 ± 3.32	0.072	0.179	9.03 ± 3.08	9.43 ± 3.49	8.97 ± 3.02	0.080	0.889
HbA1c	8.06 ± 1.74	7.87 ± 1.78	7.88 ± 1.70	0.101	0.048	7.91 ± 1.53	7.93 ± 1.79	7.73 ± 1.81	0.204	0.410
FPG category										
<7	108 (22.5)	282 (23.0)	481 (24.1)	0.656	0.943	18 (21.2)	172 (21.7)	92 (26.5)	0.185	0.092
≥7	373 (77.5)	944 (77.0)	1516 (75.9)			67 (78.8)	622 (78.3)	255 (73.5)		
HbA1c group										
<7	140 (29.1)	426 (34.7)	680 (34.1)	0.072	0.058	22 (25.9)	265 (33.4)	139 (40.1)	0.019	0.005
≥7	341 (70.9)	800 (65.3)	1317 (65.9)			63 (74.1)	529 (66.6)	208 (59.9)		
HbA1c & FPG group										
Poor	304 (63.2)	738 (60.2)	1180 (59.1)	0.266	0.257	58 (68.2)	488 (61.5)	192 (55.3)	0.090	0.008
Common	106 (22.0)	268 (21.9)	473 (23.7)			14 (16.5)	175 (22.0)	79 (22.8)		
Good	71 (14.8)	220 (17.9)	344 (17.2)			13 (15.3)	131 (16.5)	76 (21.9)		

Continuous variables were presented as the means (SD), and categorized variables as numbers (percentages).

^*^ *P*_*trend*_ values were from linear trends for one-way analysis of variance and chi-squared test.

^†^There were 8 missing values in the parental only group; 4 missing values in the sibling only group and 2 missing values in the maternal group.

### Association analysis of FH of diabetes and glycaemic control

Regarding the analysis of the association between FH of diabetes and glycaemic control, Model 1 was adjusted for age, gender, education, smoking, drinking, antidiabetic treatment, spouse with diabetes, duration of diabetes and BMI, and the results are shown in Supplementary Tables S4 and S5. Considering the missing data for physical activity (n = 1232), Model 2 included the covariates in Model 1 plus physical activity, and the results are shown in Tables 3 and 4. Overall, the fit of the two models was comparable.

**Table 3:** Multiple logistic regression analysis of FH of diabetes and glycaemic control.

Control status of HbA1c & FPG	FH group (vs. Absence)	OR (95%CI)	*P*
Poor (vs. Good)	Presence	1.366 (1.225–1.524)	<0.001
	Parental and sibling	1.411 (1.055–1.886)	0.020
	Sibling only	1.437 (1.244–1.660)	<0.001
	Parental only	1.282 (1.072–1.534)	0.007
	Bi-parental	1.688 (0.843–3.383)	0.140
	Paternal	0.918 (0.679–1.242)	0.578
	Maternal	1.449 (1.155–1.818)	0.001
Common (vs. Good)	Presence	1.275 (1.128–1.440)	<0.001
	Parental and sibling	1.274 (0.921–1.763)	0.144
	Sibling only	1.384 (1.180–1.623)	<0.001
	Parental only	1.187 (0.969–1.454)	0.099
	Bi-parental	0.962 (0.412–2.251)	0.930
	Paternal	1.000 (0.712–1.404)	0.998
	Maternal	1.343 (1.041–1.732)	0.023

Adjusted for age, gender, education, smoking, drinking, antidiabetic treatment, duration of diabetes, spouse with diabetes, BMI and physical activity.

OR: odds ratio; CI: confidence interval.

**Table 4:** Internal comparison of glycaemic control among type 2 diabetes patients with FH of diabetes.

Control status of HbA1c & FPG	Reference	FH group	OR (95%CI)	*P*
Poor (vs. Good)	Parental only	Parental and sibling	1.157 (0.830–1.612)	0.389
		Sibling only	1.156 (0.914–1.461)	0.226
	Paternal	Bi-parental	1.857 (0.864–3.989)	0.113
		Maternal	1.611 (1.106–2.347)	0.013
Common (vs. Good)	Parental only	Parental and sibling	1.087 (0.748–1.579)	0.662
		Sibling only	1.203 (0.925–1.565)	0.168
	Paternal	Bi-parental	0.980 (0.387–2.480)	0.965
		Maternal	1.381 (0.903–2.110)	0.136

Adjusted for age, gender, education, smoking, drinking, antidiabetic treatment, duration of diabetes, spouse with diabetes, BMI and physical activity.

OR: odds ratio; CI: confidence interval.

Subjects with a FH of diabetes showed a significantly higher risk of poor glycaemic control (OR 1.366, 95% CI 1.225–1.524) after adjusting for the covariates compared with those without a FH (Table 3). Patients with a “parental only”, “sibling only” and “parental and sibling” history of diabetes had a significantly higher risk of poor glycaemic control than patients without a FH, with ORs (95% CIs) of 1.282 (1.072–1.534), 1.437 (1.244–1.660) and 1.411 (1.055–1.886), respectively. Nonetheless, after Bonferroni correction (*P* value × 4), “parental only” and “sibling only” histories of diabetes remained significantly associated with a higher risk of poor glycaemic control (adjusted *P* < 0.05). In addition, compared with patients without FH, patients with a “maternal” history had a higher risk of “poor” glycaemic control (OR 1.449, 95% CI 1.155–1.818), and the association remained significant (*P* = 0.003) after Bonferroni correction (*P* value × 3). Furthermore, patients with a “sibling only” and “maternal” history also had a higher risk of “common” glycaemic control, with ORs (95% CI) of 1.384 (1.180–1.623) (adjusted *P* value by Bonferroni correction was <0.001) and 1.343 (1.041–1.732), respectively.

Compared with a “paternal” history of diabetes, a “maternal” history of diabetes resulted in a higher risk of poor glycaemic control (OR 1.611, 95% CI 1.106–2.347), and the correlation remained significant (*P* = 0.039) after Bonferroni correction (*P* value × 3) (Table 4). By contrast, no significant difference in “poor” glycaemic control was found between the “parental and sibling”, “parental only” and “sibling only” FH groups (*P* > 0.1), and there was no significant association between FH of diabetes and “common” glycaemic control among T2D patients with parental and sibling FH of diabetes.

### Stratified analyses of the relationship between FH of diabetes and glycaemic control

**Figure 1:**
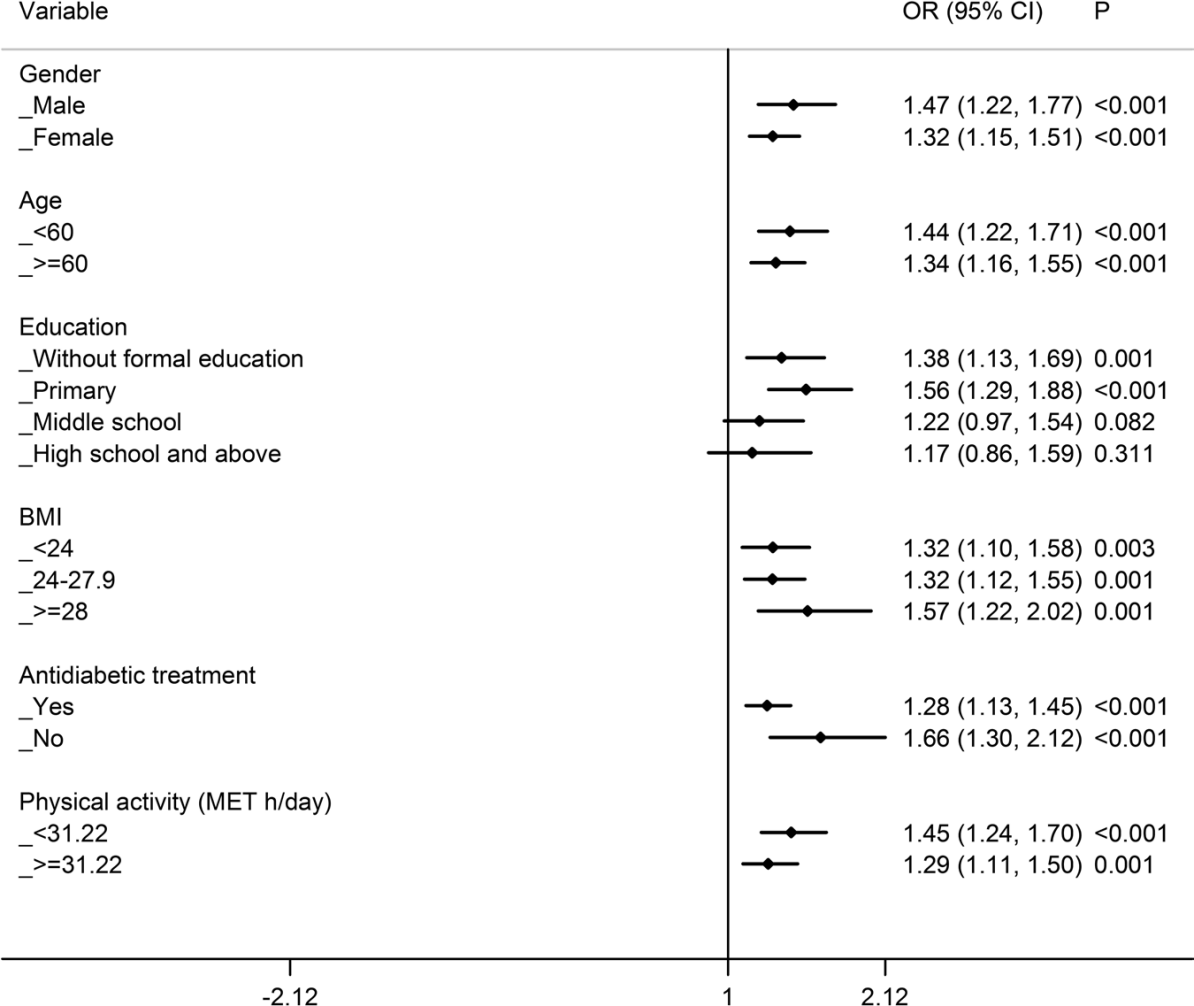
Stratified analysis of the comparison of poor glycaemic control between T2D patients with and without a FH of diabetes by age, gender, education, BMI, antidiabetic treatment and physical activity. Logistic regression was used to estimate the OR (95%CI) and each stratified analysis was adjusted for age, gender, education, BMI, antidiabetic treatment, physical activity, smoking, drinking, spouse with diabetes and duration of diabetes except the corresponding stratification factor. OR: odds ratio; CI: confidence interval.

**Figure 2:**
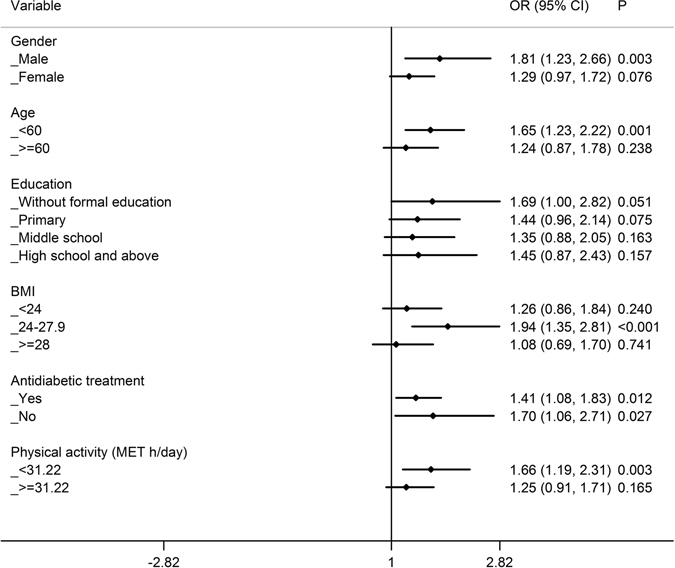
Stratified analysis of the comparison of poor glycaemic control between T2D patients with maternal history of diabetes and patients without a FH of diabetes by age, gender, education, BMI, antidiabetic treatment and physical activity. Logistic regression was used to estimate the OR (95%CI) and each stratified analysis was adjusted for age, gender, education, BMI, antidiabetic treatment, physical activity, smoking, drinking, spouse with diabetes and duration of diabetes except the corresponding stratification factor. OR: odds ratio; CI: confidence interval.

**Figure 3:**
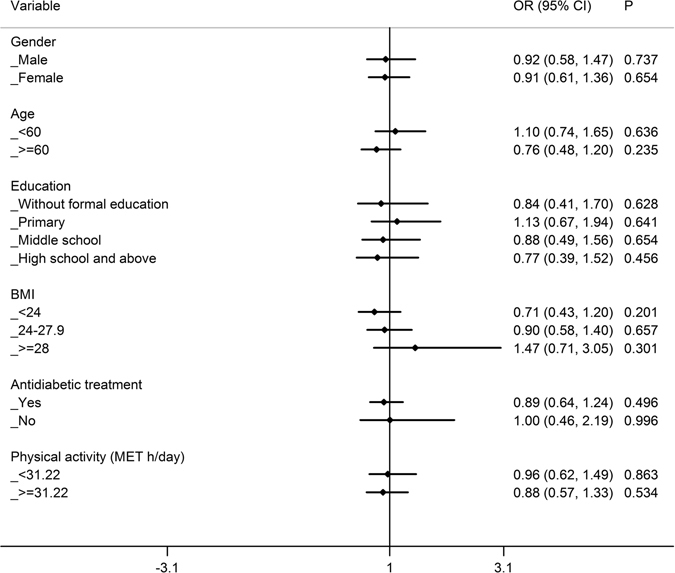
Stratified analyses of the comparison of poor glycaemic control between T2D patients with a paternal history of diabetes and patients without a FH of diabetes by age, gender, education, BMI, antidiabetic treatment and physical activity. Logistic regression was used to estimate the OR (95%CI) and each stratified analysis was adjusted for age, gender, education, BMI, antidiabetic treatment, physical activity, smoking, drinking, spouse with diabetes and duration of diabetes except the corresponding stratification factor. OR: odds ratio; CI: confidence interval.

Stratified analyses were conducted based on gender, age, education, BMI, antidiabetic treatment, and physical activity. The results of each stratified analysis, after adjusting for age, gender, education, BMI, antidiabetic treatment, physical activity, smoking, drinking, spouse with diabetes and duration of diabetes except stratification factor, are presented in the figures (Figs 1–3) as well as in Supplementary Tables S6–S10. Each of the figures contains six independent stratified analyses with adjusting for variables except stratification factor.

For each stratum of gender, age, BMI, antidiabetic treatment and physical activity, the T2D patients with a FH of diabetes had a higher risk of “poor” glycaemic control than those without a FH of diabetes (Fig. 1). In addition, among patients with formal and primary education, individuals with a FH of diabetes had a higher risk of “poor” glycaemic control than those without a FH, and the corresponding ORs (95%CIs) were 1.383 (1.133–1.688) and 1.557 (1.289–1.881), respectively. However, no difference in glycaemic control was found among patients with a relatively high level of education (middle school or high school and above).

Compared with patients without a FH of diabetes, a maternal history of diabetes was associated with a higher risk of “poor” glycaemic control among patients who were overweight (OR 1.944), aged <60 years (OR 1.653) and engaged in a low level of physical activity (OR 1.660), with *P* values < 0.05 even after Bonferroni correction (*P* value × 4) (Fig. 2). However, there was no significant difference in glycaemic control between paternal history of diabetes and no FH of diabetes in each stratum (*P* > 0.05, Fig. 3).

The homogeneity test showed that the association between a FH of diabetes (vs. without FH of diabetes) and “poor” glycaemic control was heterogeneous among patients with different education levels, as was the association between a maternal history of diabetes (vs. no FH of diabetes) and “poor” control among BMI and gender groups (*P* < 0.05).

## Discussion

Blood glucose levels are known to fluctuate over time and are influenced by several factors, including physiological metabolism, behaviour and lifestyle^36^. HbA1c reflects the average blood glucose level for the most recent three to four months and is considered an important predictive index of T2D prognostic events^37^. In this study, HbA1c and FPG were both assessed as a combined index to help evaluate glycaemic status. The results indicated that T2D patients with a FH of diabetes had a significantly higher risk of poor glycaemic control and that maternal FH in particular, rather than paternal FH, had a strong negative association with glycaemic control.

In addition, in the current study, the glycaemic control of T2D patients whose spouses had diabetes was found to be better than those whose spouses did not have diabetes. A study by Amber J^38^ suggested that shared expectations for spouse involvement could accelerate spouse’s attempts to improve T2D patients’ adherence to dietary interventions to improve glycaemic control.

The prevalence of a FH of diabetes in this study was 21.3%, which was clearly less than the rates of 30.7% among Australian T2D patients (Fremantle Diabetes Study), 34% among Danish T2D patients, and 35% among Italian T2D patients^9, 23, 25^. Here, we observed that patients with a FH of diabetes were diagnosed at an earlier age than those without a FH of diabetes. These results were consistent with a series of previous studies^9–13^. Additionally, in this study, a maternal history of diabetes had a significant adverse effect on glycaemic control in T2D patients aged <60 years, and this finding suggested that people diagnosed with T2D in early life were more likely to have worse glycaemic control^21, 22^.

Previously, studies have observed only a single glycaemic control index, either FPG or HbA1c, and have mainly investigated the influence of parental FH of diabetes. A study of 359 urban African-American T2D patients indicated a barely significant impact of a FH of diabetes on HbA1c level^24^. A longitudinal study of patients of multiple ethnicities^25^ reported that serum FPG and HbA1c remained significantly higher in T2D patients with a maternal FH of diabetes but not a paternal FH after adjusting for age, diabetes duration, and treatment type, although gender and BMI, which appeared to have an important influence on glycaemic control, were not adjusted for in that study. By contrast, a cross-sectional study of 2,113 T2D patients in Italy observed no significant influence of parental diabetes on glycaemic control^23^. Meanwhile, our current study also observed that T2D patients with a sibling who had diabetes tended to have a higher risk of “poor” or “common” glycaemic control based on the combined FPG & HbA1c index.

The results of this study suggest that maternal FH, rather than paternal FH, has a strong negative association with glycaemic control. Similarly, previous studies have indicated a stronger maternal-offspring correlation in terms of T2D compared with the paternal-offspring correlation^39–41^. These differential effects between paternal and maternal history of diabetes may contribute to the influences of maternally determined environments (intrauterine and early postnatal), genomic imprinting, and nutritional/lifestyle factors on diabetes^27–29^. Genomic imprinting can be inherited maternally rather than paternally or vice versa in several regions of the diabetes-related genome or in molecular modifications of DNA in germ-line cells^42, 43^.

Previous studies have reported that overweight and obesity were risk factors for glycaemic control in patients with diabetes^44^. In this study, the stratified analysis indicated that among overweight patients, maternal FH was a risk factor for poor glycaemic control. Boule, N. G. *et al*. reported that “high intensity exercise shows benefits with HbA1c reduction”^45^. Similarly, this study observed that in patients with a low level of physical activity (MET <31.22), but not in patients with a high level of physical activity (MET ≥ 31.22), maternal FH was a risk factor of poor glycaemic control. Meanwhile, community-based lifestyle modification programmes have been shown to be effective not only in reducing important risk factors for diabetes but also in improving blood glucose control^46, 47^. These findings imply the importance of losing weight and improving physical activity for glycaemic control in T2D patients with a FH of diabetes.

According to the findings of the current study, for community-based T2D programmes, a FH of diabetes should be routinely evaluated. This information may help determine the risk factors for poor glycaemic control in susceptible patients and may facilitate the process of designing effective family-based risk assessment and risk reduction strategies.

Our study has several strengths. First, this study used cluster sampling to survey representative T2D patients from a community-based diabetes management system and had a large sample size (20,340 subjects) to detect the influence of FH of diabetes on glycaemic control of T2D patients; these factors enabled higher statistical power and stable relevant results. Second, FPG, HbA1c and a combination of FPG and HbA1c were jointly used to assess glycaemic control, and these indices provided a comprehensive estimation of diabetes control among T2D patients. Third, the association between FH of diabetes considering various relatives and glycaemic control provided further insight into aggregate familial effect on diabetes incidence and hyperglycaemia^48^. Some limitations also existed in this study. The ‘sibling’ category might also include biologically unrelated siblings who were not identified by self-reporting; however, the proportion of siblings in this category would be low. In addition, a cross-sectional design was used, and thus causal inferences could not be made for the association of FH of diabetes with glycaemic control.

## Conclusion

The present study contributes novel evidence regarding the significant difference in the association between parental history of diabetes and glycaemic control, and low education levels and overweight could further the risks presented by a FH of diabetes. These findings have important implications for clinical interventions targeted to patients with a familial history of diabetes, particularly a maternal history.

## Electronic supplementary material

Supplemental Material (tables)
