# Supplementary material for: Familial History of Diabetes is Associated with Poor Glycaemic Control in Type 2 Diabetics: A Cross-sectional Study
Source: Sci Rep. 2017 May 3;7:1432. doi: 10.1038/s41598-017-01527-4 (PMC5431173; doi:10.1038/s41598-017-01527-4)
Supplement: Supplementary file 1 — Supplemental Material (tables) [file 41598_2017_1527_MOESM1_ESM.pdf]

Familial History of Diabetes is Associated with Poor Glycaemic Control in Type 2 Diabetics: A Cross-sectional Study

Ming Wu[1,+], Jinbo Wen[2,+], Yu Qin[1], Hailong Zhao[2], Xiaoqun Pan[1], Jian Su[1], Wencong Du[1], Enchun Pan[3], Qin Zhang[3], Ning Zhang[4], Hongyan Sheng[4], Chunlan Liu[2], Chong Shen[2,*]

1. Department of Non-communicable Chronic Disease Control, Jiangsu Provincial Center for Disease Control and Prevention, Nanjing, 210009, China

2. Department of Epidemiology, School of Public Health, Nanjing Medical University, Nanjing 211166, China

3. Department of Chronic Disease Prevention and Control, Huai'an City Center for Disease Control and Prevention, Huai'an, 223001, China

4. Changshu County Center for Disease Control and Prevention, Suzhou, 215500, China

[*]**Corresponding author**

Chong Shen, Ph.D., Associate Professor

Department of Epidemiology, School of Public Health, Nanjing Medical University, 101 Longmian Avenue, Jiangning, Nanjing, 211166 China

Tel: +86 25 86868291, Fax: +86 25 86527613, E-mail: sc@njmu.edu.cn

+these authors contributed equally to this work

Supplementary Table S1. General characteristics of participants.

Variable	Number*		Mean ± SD (%)
Age (year)	19992	-	63.35 ± 9.86
Gender	19992	Male	7843 (39.2)
		Female	12149 (60.8)
Education	19922	Without formal education	7407 (37.2)
		Primary and below	6848 (34.4)
		Middle school	3812 (19.1)
		High school and above	1855 (9.3%)
Smoking	19992	Yes	5657 (28.3)
		No	14335 (71.7)
Drinking	19932	Yes	3386 (17.0)
		Past	981 (4.9)
		No	15565 (78.1)
Antidiabetic treatment	19841	Yes	15662 (78.9)
		No	4179 (21.1)
Duration (year) †	19919	-	4.75 (2.08, 9.17)
Spouse with diabetes	18875	Yes	759 (4.0)
		No	8116 (96.0)
Familial history	19075	Presence	4070 (21.3)
		Absence	15005(78.7)
Familial history group	18875	Parental only	1226 (6.5)
		Sibling only	1997 (10.6)
		Parental and sibling	481 (2.5)
		Other relatives	166 (0.9)
		Absence	15005 (79.5)
Parental history	1226	Bi-parental	85 (6.9)
		Paternal	347 (28.3)
		Maternal	794 (64.8)
BMI (kg/m^2)	19964	-	25.33 ± 3.47
Physical activity† (MET- hour/day)	18700	-	31.20 (27.60, 36.89)
Age at diagnosis	19919	-	56.98 ± 10.55
HbA_{1c} (%)	19992	-	7.69 ± 1.81
		<7	8367 (41.9)
		≥7	11625 (58.1)
FPG (mmol/L)	19992	-	8.85 ± 3.48
		<7	6394 (32.0)
		≥7	13598 (68.0)
HbA_{1c} (%) & FPG (mmol/L)	19992	Poor	10377 (51.9)
		Common	4469 (22.4)
		Good	5146 (25.7)

Continuous variables were presented as means (SD) and categorized variables as numbers (percentages).

*There were 70 missing values for education; 60 missing values for drinking; 151 missing values for antidiabetic treatment; 73 missing values for duration; 1117 missing values for spouse with diabetes, familial history group; 917 missing values for familial history; 28 missing values for BMI, which was calculated as weight in kilograms divided by height in meters squared; 1292 missing values for physical activity; 73 missing values for age at diagnosis.

†The variable which did not comfort to normal distribution were described by their median (25 percent, 75 percent).

Supplementary Table S2. The average level of HbA1c and FPG and glycaemic control among different groups categorized by FH of parental, sibling and other relatives status.

FH of diabetes			N	HbA1c (%)	FPG (mmol/L)	HbA1c<7 (%)	FPG<7 (%)	HbA1c<7&FPG<7 (%)
parental	sibling	other relatives						
Yes	Yes	Yes	11	7.682	9.733	27.3	18.2	18.2
Yes	Yes	No	481	8.055	9.518	29.1	22.5	14.8
Yes	No	Yes	29	8.383	10.198	31.0	24.1	24.1
No	Yes	Yes	22	7.864	9.665	31.8	13.6	9.1
Yes	No	No	1226	7.871	9.276	34.7	23.0	17.9
No	Yes	No	1997	7.876	9.138	34.1	24.1	17.2
No	No	Yes	104	8.148	9.459	30.8	31.7	23.1
No	No	No	15005	7.637	8.764	44.0	34.0	27.8

Supplementary Table S3. Comparison of glycaemic control among the groups with different FH of diabetes.

FH of diabetes	N	HbA1c		FPG		HbA1c&FPG (%)			
		<7(%)	*P*	<7(%)	*P*	Poor	Common	Good	*P*
Parental and/or sibling	3704	33.6	0.738	23.5	0.109	60.0	22.9	17.1	0.172
Other relatives	104	30.8		31.7		60.6	16.3	23.1	
Both parental and/or sibling and other relatives	62	30.6		19.4		67.7	14.5	17.7	

Supplementary Table S4. The influence of diabetic FH on the glycaemic control.

Control status of HbA_{1c} & FPG	FH	FH group	OR (95%CI)	*P*
Poor	vs. Absence	Presence	1.350 (1.215-1.501)	<0.001
		Parental only	1.281 (1.075-1.527)	0.006
		Sibling only	1.410 (1.226-1.621)	<0.001
		Parental and sibling	1.405 (1.055-1.869)	0.020
	vs. Absence	Bi-parental	1.588 (0.811-3.109)	0.177
		Paternal	0.936 (0.695-1.260)	0.662
		Maternal	1.440 (1.154-1.796)	0.001
Common	vs. Absence	Presence	1.256 (1.115-1.415)	<0.001
		Parental only	1.190 (0.975-1.452)	0.087
		Sibling only	1.349 (1.156-1.575)	<0.001
		Parental and sibling	1.279 (0.928-1.763)	0.133
	vs. Absence	Bi-parental	0.979 (0.433-2.215)	0.960
		Paternal	1.001 (0.715-1.402)	0.994
		Maternal	1.344 (1.048-1.724)	0.020

Adjusted for age, gender, education, smoking, drinking, antidiabetic treatment, duration, spouse with diabetes and BMI.

OR: odds ratio; CI: confidence interval.

Supplementary Table S5. Internal comparison of the status of glycaemic control among T2D patients with different FH of diabetes.

Control status of HbA_{1c} & FPG	FH	FH group	OR (95%CI)	*P*
Poor	vs. Parental only	Parental and sibling	1.147 (0.828-1.589)	0.410
		Sibling only	1.128 (0.898-1.417)	0.300
	vs. Paternal	Bi-parental	1.691 (0.807-3.543)	0.164
		Maternal	1.567 (1.082-2,268)	0.017
Common	vs. Parental only	Parental and sibling	1.080 (0.747-1.560)	0.683
		Sibling only	1.160 (0.897-1.500)	0.258
	vs. Paternal	Bi-parental	0.990 (0.405-2.422)	0.983
		Maternal	1.373 (0.903-2.085)	0.138

Adjusted for age, gender, education, smoking, drinking, antidiabetic treatment, duration, spouse with diabetes and BMI.

OR: odds ratio; CI: confidence interval.

Supplementary Table S6. Stratified analysis on the association of diabetic FH and glycaemic control by age, gender, education, BMI, antidiabetic treatment and physical activity.

VS. Absence			Presence	
Control status of HbA1c & FPG	Stratification factors	Group	OR (95%CI)	*P*
Poor	Gender	Male	1.466 (1.216-1.767)	<0.001
		Female	1.321 (1.153-1.512)	<0.001
	Age	<60	1.444 (1.220-1.710)	<0.001
		≥60	1.341 (1.163-1.548)	<0.001
	Education	Without formal education	1.383 (1.133-1.688)	0.001
		Primary	1.557 (1.289-1.881)	<0.001
		Middle school	1.224 (0.974-1.537)	0.082
		High school and above	1.172 (0.862-1.593)	0.311
	BMI	<24	1.320 (1.102-1.582)	0.003
		24-27.9	1.316 (1.118-1.550)	0.001
		≥28	1.568 (1.217-2.020)	0.001
	Antidiabetic treatment	Yes	1.279 (1.130-1.447)	<0.001
		No	1.661 (1.301-2.122)	<0.001
	Physical activity (MET h/day)	<31.22	1.452 (1.239-1.702)	<0.001
		≥31.22	1.288 (1.107-1.497)	0.001
Common	Gender	Male	1.476 (1.201-1.815)	<0.001
		Female	1.169 (1.003-1.362)	0.045
	Age	<60	1.330 (1.099-1.608)	0.003
		≥60	1.223 (1.043-1.434)	0.013
	Education	Without formal education	1.167 (0.931-1.464)	0.181
		Primary	1.332 (1.080-1.643)	0.007
		Middle school	1.367 (1.060-1.763)	0.016
		High school and above	1.286 (0.907-1.822)	0.158
	BMI	<24	1.353 (1.111-1.649)	0.003
		24-27.9	1.251 (1.042-1.503)	0.017
		≥28	1.121 (0.829-1.515)	0.457
	Antidiabetic treatment	Yes	1.184 (1.030-1.360)	0.017
		No	1.560 (1.194-2.037)	0.001
	Physical activity (MET h/day)	<31.22	1.357 (1.135-1.622)	0.001
		≥31.22	1.200 (1.014-1.420)	0.034

Adjusted for age, gender, education, smoking, drinking, antidiabetic treatment, duration, spouse with diabetes, physical activity and BMI except stratification factor.

OR: odds ratio; CI: confidence interval.

Supplementary Table S7. Stratified analysis of the influence of parental and sibling FH on glycaemic control by age, gender, education, BMI, antidiabetic treatment and physical activity.

VS. Absence			Parental only		Sibling only		Parental and sibling	
Control status of HbA_{1c} & FPG	Stratification factors	Group	OR (95%CI)	*P*	OR (95%CI)	*P*	OR (95%CI)	*P*
Poor	Gender	Male	1.438 (1.076-1.922)	0.014	1.649 (1.266-2.150)	<0.001	1.110 (0.684-1.800)	0.673
		Female	1.200 (0.953-1.510)	0.122	1.355 (1.140-1.611)	0.001	1.588 (1.105-2.282)	0.012
	Age	<60	1.478 (1.168-1.871)	0.001	1.425 (1.096-1.853)	0.008	1.623 (1.074-2.452)	0.021
		≥60	1.096 (0.829-1.449)	0.521	1.458 (1.227-1.733)	<0.001	1.249 (0.827-1.886)	0.290
	Education	Without formal education	1.388 (0.911-2.114)	0.127	1.317 (1.043-1.663)	0.021	2.131 (1.040-4.365)	0.039
		Primary	1.367 (0.994-1.882)	0.055	1.735 (1.360-2.214)	<0.001	1.171 (0.710-1.931)	0.537
		Middle school	1.180 (0.851-1.637)	0.321	1.343 (0.950-1.900)	0.095	1.293 (0.776-2.154)	0.325
		High school and above	1.254 (0.830-1.897)	0.282	1.110 (0.681-1.812)	0.675	1.438 (0.717-2.883)	0.307
	BMI	<24	1.043 (0.772-1.409)	0.783	1.522 (1.199-1.931)	0.001	1.267 (0.809-1.984)	0.302
		24-27.9	1.521 (1.152-2.009)	0.003	1.198 (0.969-1.481)	0.095	1.388 (0.903-2.133)	0.134
		≥28	1.254 (0.856-1.837)	0.245	1.986 (1.389-2.840)	<0.001	1.977 (0.845-4.626)	0.116
	Antidiabetic treatment	Yes	1.258 (1.024-1.545)	0.029	1.311 (1.115-1.541)	0.001	1.363 (0.989-1.879)	0.059
		No	1.432 (0.964-2.128)	0.075	1.871 (1.360-2.574)	<0.001	1.388 (0.654-2.943)	0.393
	Physical activity (MET h/day)	<31.22	1.360 (1.050-1.760)	0.020	1.610 (1.303-1.991)	<0.001	1.165 (0.758-1.791)	0.485
		≥31.22	1.191 (0.927-1.530)	0.171	1.307 (1.073-1.592)	0.008	1.600 (1.079-2.373)	0.019
Common	Gender	Male	1.485 (1.079-2.044)	0.015	1.644 (1.231-2.196)	0.001	1.263 (0.743-2.146)	0.388
		Female	0.998 (0.764-1.305)	0.991	1.273 (1.051-1.542)	0.014	1.247 (0.826-1.883)	0.294
	Age	<60	1.191 (0.909-1.561)	0.205	1.668 (1.254-2.218)	<0.001	1.339 (0.838-2.139)	0.222
		≥60	1.158 (0.853-1.573)	0.348	1.265 (1.044-1.533)	0.016	1.209 (0.770-1.899)	0.410
	Education	Without formal education	1.172 (0.723-1.899)	0.520	1.106 (0.848-1.443)	0.456	2.051 (0.947-4.440)	0.068
		Primary	1.188 (0.828-1.703)	0.350	1.492 (1.141-1.949)	0.003	1.101 (0.630-1.922)	0.735
		Middle school	1.315 (0.912-1.896)	0.142	1.634 (1.123-2.377)	0.010	1.182 (0.660-2.116)	0.575
		High school and above	1.124 (0.692-1.827)	0.637	1.667 (0.991-2.804)	0.054	1.161 (0.512-2.632)	0.720
	BMI	<24	1.235 (0.892-1.711)	0.203	1.444 (1.114-1.871)	0.005	1.443 (0.891-2.337)	0.136
		24-27.9	1.221 (0.888-1.680)	0.219	1.299 (1.030-1.640)	0.027	1.233 (0.760-2.000)	0.397
		≥28	1.003 (0.636-1.584)	0.988	1.473 (0.975-2.223)	0.066	0.650 (0.200-2.116)	0.474
	Antidiabetic treatment	Yes	1.182 (0.938-1.491)	0.157	1.245 (1.040-1.491)	0.017	1.210 (0.844-1.735)	0.300
		No	1.082 (0.669-1.751)	0.747	1.830 (1.308-2.560)	<0.001	1.631 (0.751-3.539)	0.216
	Physical activity (MET h/day)	<31.22	1.246 (0.929-1.672)	0.142	1.530 (1.210-1.934)	<0.001	1.286 (0.800-2.068)	0.299
		≥31.22	1.117 (0.843-1.480)	0.441	1.273 (1.024-1.582)	0.030	1.241 (0.796-1.937)	0.341

Adjusted for age, gender, education, smoking, drinking, antidiabetic treatment, duration, spouse with diabetes, physical activity and BMI except stratification factor.

OR: odds ratio; CI: confidence interval.

Supplementary Table S8. Stratified analysis on the comparison of glycaemic control between parental and sibling history of diabetes.

VS. Parental only			Parental and sibling		Sibling only	
Control status of HbA_{1c} & FPG	Stratification factors	Group	OR (95%CI)	*P*	OR (95%CI)	*P*
Poor	Gender	Male	0.798 (0.461-1.382)	0.420	1.167 (0.780-1.747)	0.452
		Female	1.373 (0.906-2.080)	0.136	1.149 (0.860-1.534)	0.348
	Age	<60	1.114 (0.699-1.775)	0.649	0.938 (0.667-1.318)	0.711
		≥60	1.200 (0.740-1.948)	0.460	1.278 (0.928-1.759)	0.134
	Education	Without formal education	1.555 (0.679-3.559)	0.296	0.969 (0.593-1.584)	0.901
		Primary	0.926 (0.518-1.654)	0.795	1.317 (0.882-1.966)	0.178
		Middle school	1.116 (0.624-1.996)	0.711	1.131 (0.713-1.795)	0.601
		High school and above	1.179 (0.534-2.604)	0.684	0.988 (0.522-1.869)	0.971
	BMI	<24	1.249 (0.750-2.080)	0.392	1.471 (1.008-2.146)	0.045
		24-27.9	0.947 (0.573-1.566)	0.833	0.778 (0.542-1.116)	0.173
		≥28	1.494 (0.576-3.870)	0.409	1.835 (1.029-3.271)	0.040
	Antidiabetic treatment	Yes	1.149 (0.791-1.667)	0.466	1.080 (0.829-1.407)	0.569
		No	0.916 (0.383-2.190)	0.843	1.353 (0.761-2.404)	0.303
	Physical activity (MET h/day)	<31.22	0.917 (0.560-1.501)	0.730	1.331 (0.935-1.894)	0.113
		≥31.22	1.411 (0.900-2.212)	0.133	1.060 (0.774-1.451)	0.717
Common	Gender	Male	0.820 (0.448-1.501)	0.520	1.076 (0.692-1.673)	0.745
		Female	1.257 (0.780-2.025)	0.348	1.320 (0.947-1.839)	0.102
	Age	<60	1.118 (0.658-1.899)	0.681	1.343 (0.919-1.961)	0.127
		≥60	1.039 (0.608-1.775)	0.888	1.108 (0.776-1.580)	0.573
	Education	Without formal education	1.635 (0.661-4.048)	0.287	0.933 (0.532-1.634)	0.807
		Primary	0.956 (0.499-1.832)	0.892	1.238 (0.793-1.933)	0.347
		Middle school	0.854 (0.437-1.667)	0.643	1.157 (0.692-1.936)	0.578
		High school and above	1.001 (0.400-2.505)	0.998	1.568 (0.785-3.131)	0.203
	BMI	<24	1.230 (0.707-2.139)	0.464	1.211 (0.802-1.827)	0.362
		24-27.9	1.004 (0.567-1.776)	0.990	1.074 (0.714-1.615)	0.731
		≥28	0.617 (0.172-2.217)	0.460	1.597 (0.820-3.109)	0.169
	Antidiabetic treatment	Yes	1.044 (0.687-1.587)	0.841	1.108 (0.823-1.491)	0.499
		No	1.396 (0.561-3.472)	0.473	1.577 (0.834-2.981)	0.161
	Physical activity (MET h/day)	<31.22	1.059 (0.612-1.833)	0.837	1.390 (0.936-2.065)	0.103
		≥31.22	1.144 (0.687-1.907)	0.605	1.105 (0.776-1.574)	0.578

Adjusted for age, gender, education, smoking, drinking, antidiabetic treatment, duration, spouse with diabetes, physical activity and BMI except stratification factor.

OR: odds ratio; CI: confidence interval.

Supplementary Table S9. Stratified analysis of the influence of parental diabetes on glycaemic control by age, gender, education, BMI, antidiabetic treatment and physical activity.

VS. Absence			Bi-parental		Paternal		Maternal	
Control status of HbA_{1c} & FPG	Stratification factors	Group	OR (95%CI)	*P*	OR (95%CI)	*P*	OR (95%CI)	*P*
Poor	Gender	Male	1.730 (0.617-4.855)	0.298	0.924 (0.581-1.467)	0.737	1.809 (1.231-2.660)	0.003
		Female	1.630 (0.639-4.159)	0.306	0.913 (0.612-1.362)	0.654	1.294 (0.974-1.720)	0.076
	Age	<60	1.628 (0.728-3.638)	0.235	1.103 (0.735-1.654)	0.636	1.653 (1.232-2.219)	0.001
		≥60	2.439 (0.537-11.065)	0.248	0.757 (0.479-1.198)	0.235	1.241 (0.867-1.778)	0.238
	Education	Without formal education	NV	NV	0.840 (0.415-1.700)	0.628	1.686 (0.997-2.851)	0.051
		Primary	2.294 (0.496-10.596)	0.288	1.135 (0.666-1.936)	0.641	1.438 (0.964-2.145)	0.075
		Middle school	1.012 (0.402-2.549)	0.981	0.877 (0.494-1.556)	0.654	1.349 (0.885-2.054)	0.163
		High school and above	2.785 (0.529-14.664)	0.227	0.772 (0.391-1.524)	0.456	1.451 (0.867-2.429)	0.157
	BMI	<24	0.830 (0.287-2.394)	0.730	0.714 (0.426-1.197)	0.201	1.256 (0.859-1.837)	0.240
		24-27.9	2.143 (0.677-6.779)	0.195	0.905 (0.584-1.403)	0.657	1.944 (1.347-2.807)	<0.001
		≥28	3.091 (0.668-14.309)	0.149	1.469 (0.708-3.045)	0.301	1.079 (0.686-1.698)	0.741
	Antidiabetic treatment	Yes	2.026 (0.860-4.769)	0.106	0.891 (0.638-1.243)	0.496	1.406 (1.079-1.831)	0.012
		No	1.281 (0.296-5.535)	0.740	1.002 (0.458-2.190)	0.996	1.698 (1.062-2.715)	0.027
	Physical activity (MET h/day)	<31.22	1.162 (0.489-2.759)	0.734	0.963 (0.624-1.486)	0.863	1.660 (1.192-2.311)	0.003
		≥31.22	2.773 (0.806-9.542)	0.106	0.875 (0.574-1.333)	0.534	1.248 (0.913-1.707)	0.165
Common	Gender	Male	1.400 (0.433-4.523)	0.574	1.039 (0.620-1.741)	0.883	1.892 (1.246-2.874)	0.003
		Female	0.600 (0.164-2.199)	0.440	0.962 (0.611-1.515)	0.866	1.067 (0.766-1.486)	0.701
	Age	<60	0.563 (0.182-1.744)	0.319	1.039 (0.655-1.648)	0.870	1.347 (0.964-1.883)	0.081
		≥60	2.429 (0.487-12.121)	0.279	0.905 (0.548-1.497)	0.699	1.291 (0.872-1.910)	0.203
	Education	Without formal education	NV	NV	1.094 (0.504-2.371)	0.821	1.238 (0.674-2.274)	0.492
		Primary	1.459 (0.255-8.363)	0.671	1.063 (0.582-1.942)	0.842	1.257 (0.800-1.973)	0.321
		Middle school	0.639 (0.195-2.102)	0.461	1.042 (0.549-1.977)	0.901	1.600 (1.010-2.533)	0.045
		High school and above	1.812 (0.265-12.383)	0.545	0.911 (0.413-2.011)	0.818	1.228 (0.671-2.246)	0.506
	BMI	<24	0.532 (0.139-2.042)	0.358	1.226 (0.719-2.090)	0.455	1.348 (0.891-2.040)	0.157
		24-27.9	1.127 (0.285-4.457)	0.865	0.716 (0.421-1.217)	0.217	1.689 (1.122-2.544)	0.012
		≥28	1.688 (0.272-10.484)	0.574	1.271 (0.546-2.959)	0.578	0.884 (0.510-1.531)	0.659
	Antidiabetic treatment	Yes	1.267 (0.472-3.405)	0.639	0.976 (0.670-1.422)	0.899	1.331 (0.991-1.788)	0.058
		No	NV	NV	1.077 (0.456-2.540)	0.866	1.216 (0.685-2.157)	0.505
	Physical activity (MET h/day)	<31.22	1.087 (0.399-2.958)	0.871	1.055 (0.645-1.726)	0.831	1.445 (0.992-2.104)	0.055
		≥31.22	0.730 (0.143-3.721)	0.705	0.953 (0.595-1.526)	0.840	1.235 (0.873-1.748)	0.233

Adjusted for age, gender, education, smoking, drinking, antidiabetic treatment, duration, spouse with diabetes, physical activity and BMI except stratification factor.

OR: odds ratio; CI: confidence interval; NV: not valid.

Supplementary Table S10. Stratified analysis on the comparison of glycaemic control between paternal and maternal diabetes by age, gender, education, BMI, antidiabetic treatment and physical activity.

VS. Paternal			Bi-parental		Maternal	
Control status of HbA1c & FPG	Stratification factors	Group	OR (95%CI)	*P*	OR (95%CI)	*P*
Poor	Gender	Male	1.826 (0.568-5.866)	0.312	1.983 (1.081-3.638)	0.027
		Female	1.790 (0.640-5.003)	0.267	1.417 (0.870-2.309)	0.161
	Age	<60	1.542 (0.598-3.977)	0.370	1.539 (0.919-2.579)	0.101
		≥60	3.685 (0.764-17.787)	0.104	1.652 (0.938-2.907)	0.082
	Education	Without formal education	NV	NV	1.723 (0.663-4.479)	0.264
		Primary	2.214 (0.426-11.492)	0.344	1.323 (0.691-2.532)	0.398
		Middle school	1.114 (0.359-3.459)	0.852	1.738 (0.830-3.642)	0.143
		High school and above	3.988 (0.581-27.381)	0.159	2.092 (0.855-5.120)	0.106
	BMI	<24	1.361 (0.420-4.413)	0.608	1.824 (0.984-3.381)	0.056
		24-27.9	2.331 (0.631-8.609)	0.204	2.266 (1.248-4.112)	0.007
		≥28	2.150 (0.337-13.711)	0.418	0.676 (0.261-1.752)	0.420
	Antidiabetic treatment	Yes	2.261 (0.896-5.707)	0.084	1.595 (1.048-2.427)	0.029
		No	1.096 (0.159-7.535)	0.926	1.592 (0.606-4.185)	0.345
	MET (h/day)	<31.22	1.213 (0.450-3.272)	0.703	1.762 (1.016-3.058)	0.044
		≥31.22	3.219 (0.870-11.912)	0.080	1.449 (0.864-2.432)	0.160
Common	Gender	Male	1.372 (0.368-5.112)	0.637	1.887 (0.972-3.662)	0.060
		Female	0.602 (0.150-2.426)	0.476	1.097 (0.624-1.928)	0.747
	Age	<60	0.588 (0.165-2.092)	0.412	1.362 (0.760-2.440)	0.299
		≥60	2.663 (0.491-14.431)	0.256	1.408 (0.749-2.647)	0.289
	Education	Without formal education	NV	NV	1.087 (0.380-3.112)	0.876
		Primary	1.769 (0.266-11.763)	0.555	1.319 (0.625-2.785)	0.467
		Middle school	0.620 (0.152-2.534)	0.506	1.804 (0.787-4.135)	0.163
		High school and above	2.304 (0.264-20.109)	0.450	1.519 (0.555-4.160)	0.416
	BMI	<24	0.503 (0.116-2.179)	0.358	1.101 (0.565-2.145)	0.778
		24-27.9	1.417 (0.299-6.709)	0.661	2.495 (1.246-4.999)	0.010
		≥28	1.177 (0.136-10.150)	0.882	0.683 (0.234-1.996)	0.486
	Antidiabetic treatment	Yes	1.266 (0.436-3.676)	0.665	1.368 (0.854-2.191)	0.192
		No	NV	NV	1.307 (0.419-4.084)	0.645
	MET (h/day)	<31.22	1.015 (0.320-3.216)	0.980	1.416 (0.757-2.648)	0.276

≥31.22	0.836 (0.152-4.610)	0.837	1.343 (0.749-2.408)	0.322

Adjusted for age, gender, education, smoking, drinking, antidiabetic treatment, duration, spouse with diabetes , physical activity and BMI except stratification factor.

OR: odds ratio; CI: confidence interval; NV: not valid.
